# Metrnl and Cardiomyopathies: From Molecular Mechanisms to Therapeutic Insights

**DOI:** 10.1111/jcmm.70371

**Published:** 2025-01-23

**Authors:** Miaomiao Xu, Xiaoguang Liu, Liming Lu, Zhaowei Li

**Affiliations:** ^1^ School of Physical Education and Health Guangzhou University of Chinese Medicine Guangzhou Guangdong China; ^2^ South China Research Center for Acupuncture and Moxibustion, Medical College of Acu‐Moxi and Rehabilitation Guangzhou University of Chinese Medicine Guangzhou Guangdong China; ^3^ College of Sports and Health Guangzhou Sport University Guangzhou Guangdong China

**Keywords:** adipokine, cardiokine, cardiomyopathies, Metrnl, myokine

## Abstract

Cardiomyopathies, a diverse group of diseases affecting the heart muscle, continue to pose significant clinical challenges due to their complex aetiologies and limited treatment options targeting underlying genetic and molecular dysregulations. Emerging evidence indicates that Metrnl, a myokine, adipokine and cardiokine, plays a significant role in the pathogenesis of various cardiomyopathies. Therefore, the objective of this review is to examine the role and mechanism of Metrnl in various cardiomyopathies, with the expectation of providing new insights for the treatment of these diseases.

## 1 Introduction

Cardiomyopathies are a heterogeneous group of diseases that primarily affect the heart muscle, leading to both structural and functional abnormalities [1, 2]. Cardiomyopathies can lead to various clinical manifestations, such as heart failure (HF), arrhythmias and sudden cardiac death [3, 4, 5]. According to the 2024 report by the American Heart Association (AHA), cardiomyopathy was the primary diagnosis in 14,270 hospital admissions and was identified in a total of 1,083,430 cases, as per the 2020 Healthcare Cost and Utilization Project (HCUP) data [6]. Cardiomyopathies can be divided into two major groups, primary cardiomyopathies and secondary cardiomyopathies. Primary cardiomyopathies (genetic, nongenetic, acquired) are diseases that are predominantly or solely confined to the heart muscle and are relatively rare compared to other heart conditions [7, 8]. Examples include hypertrophic cardiomyopathy, dilated cardiomyopathy, primary restrictive nonhypertrophied cardiomyopathy, myocarditis and Takotsubo cardiomyopathy [9, 10]. In contrast, secondary cardiomyopathies describe conditions in which cardiac involvement occurs as part of a systemic condition or “heart muscle disease resulting from an extracardiac vascular cause is known as secondary cardiomyopathy.” These conditions include ischemic heart disease, hypertension, valvular heart disease, congenital heart disease, neuromuscular disorders, endocrine disorders and other systemic diseases [11]. Additionally, it should be noted that this classification isn't 100% clear‐cut, and in certain cases, it may overlap. Most therapeutic interventions for cardiomyopathy involve the use of pharmacological agents originally developed for HF, aiming to alleviate symptoms and prevent adverse outcomes. Despite advances in treatment, there remains a significant gap in therapies that specifically target the underlying genetic and molecular dysregulations of cardiomyopathies [12, 13]. There is an increasing need for a deeper understanding of the intrinsic pathophysiological mechanisms underlying cardiomyopathies. This need is driven by the demand for the development of therapeutic modalities that are not only scientifically sound but also exhibit a higher degree of precision and rationale in their treatment approach. Moreover, there is a growing interest in finding more therapies targeted towards the specific mechanisms of diseases rather than solely focusing on alleviating the symptoms related to them.

Myokines, adipocytokines and cardiokines are types of molecules secreted by skeletal muscle, adipose tissue and the myocardium. Certain myokines, adipocytokines, and cardiokines have been linked to the pathogenesis of specific diseases, including acute myocardial infarction [14, 15], myocardial ischemia/reperfusion injury [16], HF [17, 18], cardiac hypertrophy [19, 20], diabetic cardiomyopathy and others [21, 22]. Metrnl, also known as meteorin‐like protein, subfatin, cometin, Meteorin‐β or interleukin (IL)‐41, is encoded by the Metrnl. It is secreted and shares structural similarities with the neurotrophic factor Meteorin [23, 24]. It has been identified as a myokine and adipocytokine, which is expressed in various tissues, including skeletal muscle, adipose tissue and the myocardium. Its expression levels can change in response to certain stimuli, such as exercise, cold exposure or pathological stimuli [25, 26, 27].

During the past decade, an extensive corpus of scholarly inquiry has progressively revealed the multifaceted role of Metrnl in the aetiology of a diverse array of pathological conditions. Notably, the protein's regulatory capacity has been implicated in the pathophysiology of muscle atrophy [28], fulminant hepatitis [29], atherosclerosis [30], allergic asthma [31], atopic dermatitis [32], chronic obstructive pulmonary disease (COPD) and other conditions [33]. Recently, there has been growing interest among researchers in Metrnl's potential cardioprotective effects on cardiomyopathies (CMs). For instance, Hu et al. explored Metrnl's cardioprotective effect in doxorubicin (DOX)‐induced cardiotoxicity [34]. Rupérez et al. investigated Metrnl's protective effects in isoproterenol‐induced cardiac hypertrophy and ageing [35]. However, a comprehensive review summarising the role of Metrnl in CMs is lacking. In this review, we focus on the origin, expression pattern, and cellular and molecular mechanisms of Metrnl's cardioprotective effects.

## 2 Metrnl Bioinformatics and Structure

Bioinformatics predicts that the Metrnl protein consists of 311 amino acids. It includes an NH2‐terminal signal peptide of 45 amino acids, indicating that the mature, secreted form of the protein is composed of 266 amino acids [36, 37]. Metrnl comprises two primary functional domains: a CUB domain and an NTR domain, connected by a hinge/loop region. This structure is homologous to the neurotrophin Metrn [38]. The CUB domain and NTR domain are critical for receptor binding and functional interaction. This structure allows Metrnl to interact effectively with its receptors, such as the KIT receptor, influencing various physiological processes [38].

### 2.1 The Different Roles of Metrnl as a Cytokine

#### 2.1.1 Myokine

Myokines are cytokines and peptides produced and released by skeletal muscle cells in response to muscle contraction or other stimuli. They function in a hormone‐like manner, exerting specific endocrine effects on other organs and tissues, including heart [39, 40]. Myokines have emerged as promising targets for the treatment of cardiomyopathies, as evidenced by recent research. Musclin, a member of the myokine family, plays a crucial role in cardiac health. In a mouse model of HF induced by pathological overload, reduced levels of skeletal muscle Musclin have been shown to exacerbate cardiac dysfunction and fibrosis. Conversely, mice with overexpressed Musclin exhibit a mitigation of these cardiac abnormalities, suggesting that modulating Musclin levels could be a potential therapeutic strategy for managing cardiomyopathies [41].

Metrnl was first reported as secreted proteins in 2009, which is homologous to Metrn [42, 43]. Metrnl is a myokine that plays significant roles in muscle metabolism, inflammation regulation and systemic energy homeostasis. It is secreted by muscle cells during exercise and has been shown to have various beneficial effects on glucose metabolism, such as improving insulin sensitivity and enhancing glucose uptake in muscle cells, and immune function [44, 45].

Metabolic and inflammatory regulation. Moderate‐intensity physical activity enhances the release of Metrnl, which, in turn, mitigates inflammation and pyroptosis [46]. It mitigates lipid‐mediaterd inflammatory responses and enhances insulin sensitivity in murine skeletal muscle through AMP‐activated protein kinase (AMPK) or peroxisome proliferator‐activated receptor‐δ (PPAR‐δ)‐dependent mechanisms [47]. Lee et al. [48] demonstrated that intraperitoneal administration of recombinant Metrnl has demonstrated efficacy in increasing glucose uptake via AMPKα2 in murine models with obesity or type 2 diabetes mellitus.

Skeletal muscle regeneration. Metrnl is implicated in the process of skeletal muscle regeneration: mice that are genetically deficient in Metrnl or have Metrnl specifically knocked out in their macrophages exhibit impaired muscle repair [49]. Consistent with these findings, Metrnl, as an injectable peptide, has the potential to enhance the regeneration of aged skeletal muscle. Metrnl counteracts a pro‐fibrotic gene programme by inducing TNFα‐mediated apoptosis of fibro/adipogenic progenitor (FAP) cells, thereby enhancing immune responsiveness during muscle regeneration [28].

Metrnl, a myokine, is pivotal in muscle metabolism, inflammation regulation and the maintenance of systemic energy balance. Its functions are integral to the pathophysiology of cardiomyopathies, impacting cardiac function, insulin sensitivity and systemic inflammation—factors that are essential in both the development and progression of these heart conditions.

#### 2.1.2 Adipocytokines

Adipocytokines, also referred to as adipokines, are a notable group of proteins and peptides secreted by adipose tissue [50, 51]. It is not only implicated in metabolic diseases but also serves as a therapeutic agent in the treatment of cardiomyopathies [52, 53]. Metrnl, as an adipokine, is expressed in adipose tissue and exerts various effects on metabolic regulation, inflammation, adipose tissue function [54, 55] and cardiomyopathies [42].

Effects on insulin sensitivity. Metrnl improves insulin sensitivity through the PPARγ pathway. Adipocyte‐specific knockout of Metrnl exacerbates insulin resistance, whereas its overexpression prevents this effect without altering body weight and adipose content [56].

Effects on inflammation. Metrnl has been identified as an adipocytokine that mediates its effects through the activation of macrophages, exhibiting properties that counteract inflammation. Metrnl exhibits anti‐inflammatory properties through the activation of macrophages. Rao et al. [57] found that Metrnl modulates the immune response by inducing eosinophil‐mediated enhancement of IL‐4 transcription, which plays a crucial role in the alternative activation of adipose tissue macrophages. Moreover, the inhibition of Metrnl signalling in vivo significantly dampens the alternative activation of macrophages and the expression of thermogenic genes induced by prolonged exposure to cold conditions [57].

Effects on adipose tissue function. Metrnl also plays an important role in adipose tissue function. It's expression decreases during adipogenesis (i.e., adipocyte differentiation) and inhibits further adipocyte differentiation, leading to adipocyte hypertrophy (i.e., increased cell size) in humans [58].

In conclusion, Metrnl, as an adipokine, plays a pivotal role in regulating insulin sensitivity, lipid metabolism and inflammation. It's effects on adipocyte differentiation and function, as well as its potential for treating metabolic diseases and cardiovascular conditions, render it a significant target for therapeutic interventions.

#### 2.1.3 Cardiokine

Cardiokines are proteins secreted by the heart that play crucial roles in maintaining heart function, responding to myocardial damage and influencing other organs. They have potential as therapeutic targets for various cardiovascular diseases [59, 60, 61]. Metrnl functions as a myokine, adipokine and cardiokine, highlighting its versatile roles in various tissues. As a cardiokine, it has cardioprotective effects, particularly in helping cardiac hypertrophy, dysfunction [62], myocardial ischemia/reperfusion injury [63], postinfarction recovery [64]. This review will further discuss Metrnl's protective role in heart disease.

### 2.2 Expression Pattern of Metrnl in Cardiomyopathies

Metrnl is expressed in a range of cell types, including adipocytes, skeletal muscle cells, astrocytes, macrophages and fibroblasts. Notably, its expression is significantly altered in the heart under different pathological conditions in both rodent and human models, suggesting a potential role in the pathophysiology of these conditions.

#### 2.2.1 Elevated Metrnl Expression in Cardiac Tissue

**FIGURE 1:**
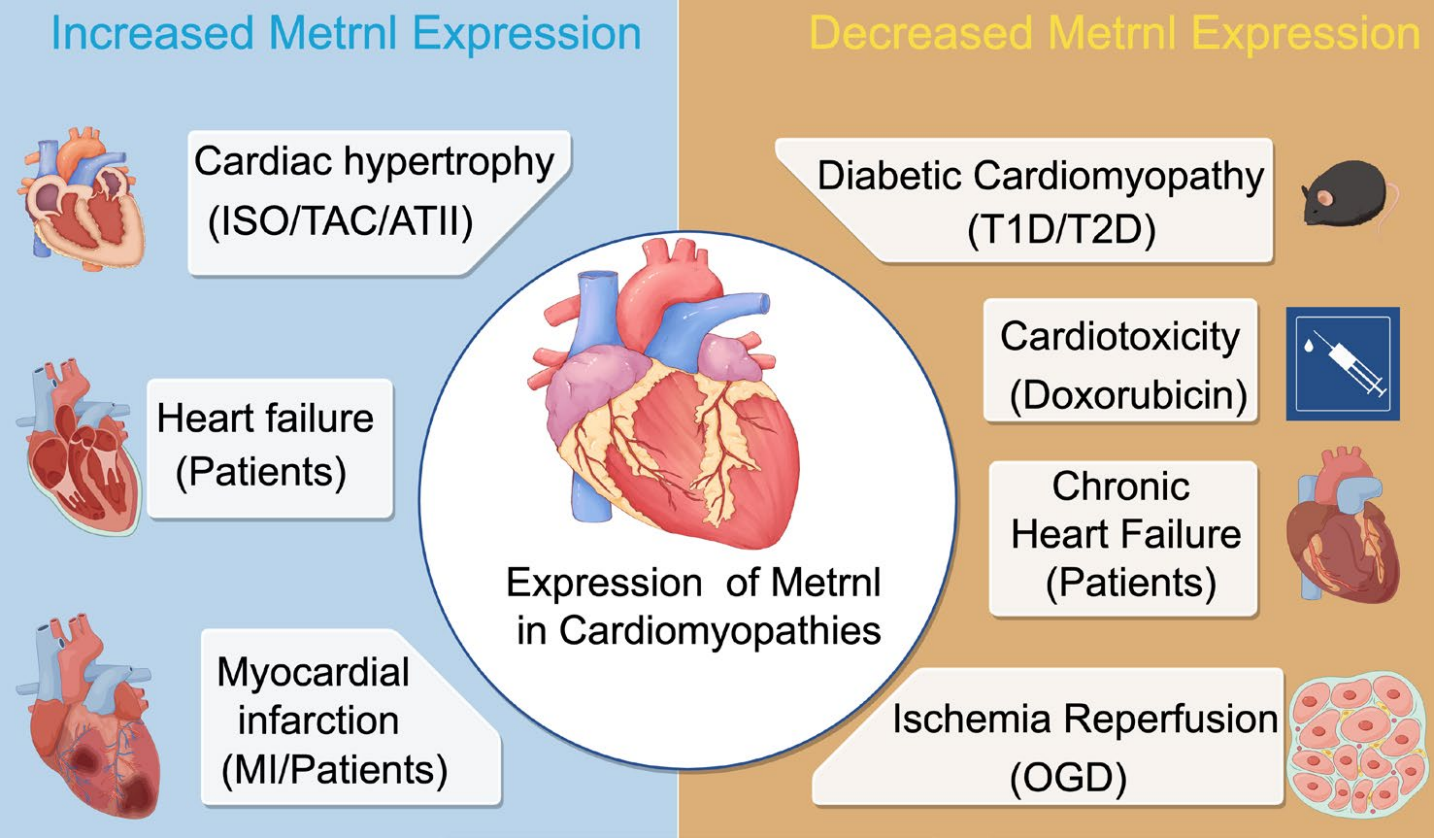
Graphical summary of the expression pattern of Metrnl in cardiomyopathies. Figure was prepared with figdraw.com. ATII, angiotensin II; ISO, isoproterenol; MI, myocardial infarction; OGD, oxygen–glucose deprivation; TAC, transverse aortic constriction; T1D, type 1 diabetic; T2D, type 2 diabetic.

Numerous studies have reported Metrnl expression in cardiac tissue across various species, including rats, mice and humans. The levels of Metrnl expression in the heart are markedly influenced by the pathological state. In healthy human hearts, Metrnl expression is lower compared to that in adipose and skeletal tissues. However, the highest levels of Metrnl expression in the heart have been observed relative to those in brown adipose tissue (BAT), inguinal white adipose tissue (iWAT), epididymal white adipose tissue (eWAT), liver and skeletal muscle in adult mice (Figure 1) [34, 35].

#### 2.2.2 Increased Metrnl Expression in Cardiac Hypertrophy, Myocardial Infarction, and Heart Failure

Cardiac hypertrophy, characterised by the enlargement of the heart muscle, typically arises as an adaptive response to increased workload or pathological conditions [65, 66]. It can be induced by various factors, including hypertension, myocardial infarction and valvular disease, and is categorised into physiological and pathological types based on the underlying aetiology and clinical outcomes [67, 68]. In models of cardiac hypertrophy, including isoproterenol (ISO), transverse aortic constriction (TAC) and angiotensin II (ATII), Metrnl expression was significantly elevated compared to control groups [35]. Following MI in mice, Metrnl expression increased, and a similar pattern was observed in patients with acute MI.

Myocardial infarction (MI), commonly known as a heart attack, is a serious condition characterised by the obstruction of blood flow to the heart muscle, resulting in tissue damage [69, 70]. This obstruction is usually caused by atherosclerotic plaque buildup in the coronary arteries, leading to ischemia and necrosis of the myocardial tissue [71, 72]. Following myocardial infarction, a notable increase in Metrnl expression was observed in the myocardium compared to sham controls. Additionally, elevated Metrnl expression was observed in the cardiac tissue of acute MI patients, with RT‐PCR and single‐cell RNA sequencing identifying monocytes and macrophages as the primary sources of Metrnl in the MI‐affected heart [64].

Heart failure (HF) is a multifaceted clinical syndrome characterised by the heart's inability to pump sufficient blood to meet the metabolic demands of the body [73, 74]. This condition can result from multiple aetiologies, including coronary artery disease, hypertension, valvular disorders and cardiomyopathies [75, 76]. Metrnl expression levels were significantly upregulated in HF patients compared to healthy controls [35].

#### 2.2.3 Decreased Metrnl Expression in Cardiotoxicity, Chronic Heart Failure, Diabetic Cardiomyopathy, and Ischemia/Reperfusion Injury

Metrnl expression in cardiac muscle was significantly reduced following doxorubicin administration in murine models, as confirmed by western blot and ELISA analyses [34]. Doxorubicin, a potent chemotherapeutic agent, is effective against a range of malignancies but is also associated with significant cardiotoxicity, potentially leading to irreversible myocardial damage and HF [77, 78].

Chronic heart failure (CHF) is a chronic condition characterised by the heart's impaired ability to maintain adequate circulatory output, resulting in symptoms such as fatigue, dyspnea and fluid retention [79, 80]. CHF may arise from various underlying conditions, including coronary artery disease, hypertension and cardiomyopathies [81, 82]. Reduced serum levels of Metrnl in elderly CHF patients have been correlated with poorer clinical outcomes and greater severity of cardiac dysfunction [83].

Diabetic cardiomyopathy is a specific cardiac pathology associated with diabetes mellitus, characterised by myocardial dysfunction independent of coronary artery disease or hypertension [84, 85]. This condition is characterised by structural and functional abnormalities in the myocardium, leading to HF [86, 87]. In streptozotocin (STZ)‐induced type 1 diabetic (T1D) and db/db type 2 diabetic (T2D) murine models, Metrnl expression in both cardiac tissue and plasma was significantly reduced [88]. Additionally, chronic hyperglycaemia was found to markedly suppress Metrnl mRNA expression in cardiomyocytes, but not in cardiac endothelial cells or fibroblasts [88].

Myocardial ischemia/reperfusion (I/R) injury, defined as the damage incurred upon the restoration of blood supply to the heart following a period of ischemia, presents a paradoxical challenge in myocardial salvage [89, 90]. While reperfusion is crucial for restoring oxygen and nutrients, it may exacerbate tissue injury. In the myocardial ischemia/reperfusion (MI/R) injury H9C2 cell model, significant reduction in Metrnl expression was observed [63]. The H9C2 cell line, derived from rat cardiac tissue, is widely utilised in research to model MI/R injury, thereby facilitating the exploration of cellular and molecular mechanisms as well as potential therapeutic interventions.

## 3 Protective Mechanisms of Metrnl in Cardiomyopathies

Metrnl has been shown to have protective effects in various cardiomyopathies through several mechanisms. These mechanisms include improving cardiotoxicity, alleviating diabetic cardiomyopathy, promoting angiogenesis in myocardial infarction, reducing myocardial hypertrophy and protecting against myocardial ischemia/reperfusion injury.

### 3.1 Metrnl Improves Cardiotoxicity

**FIGURE 2:**
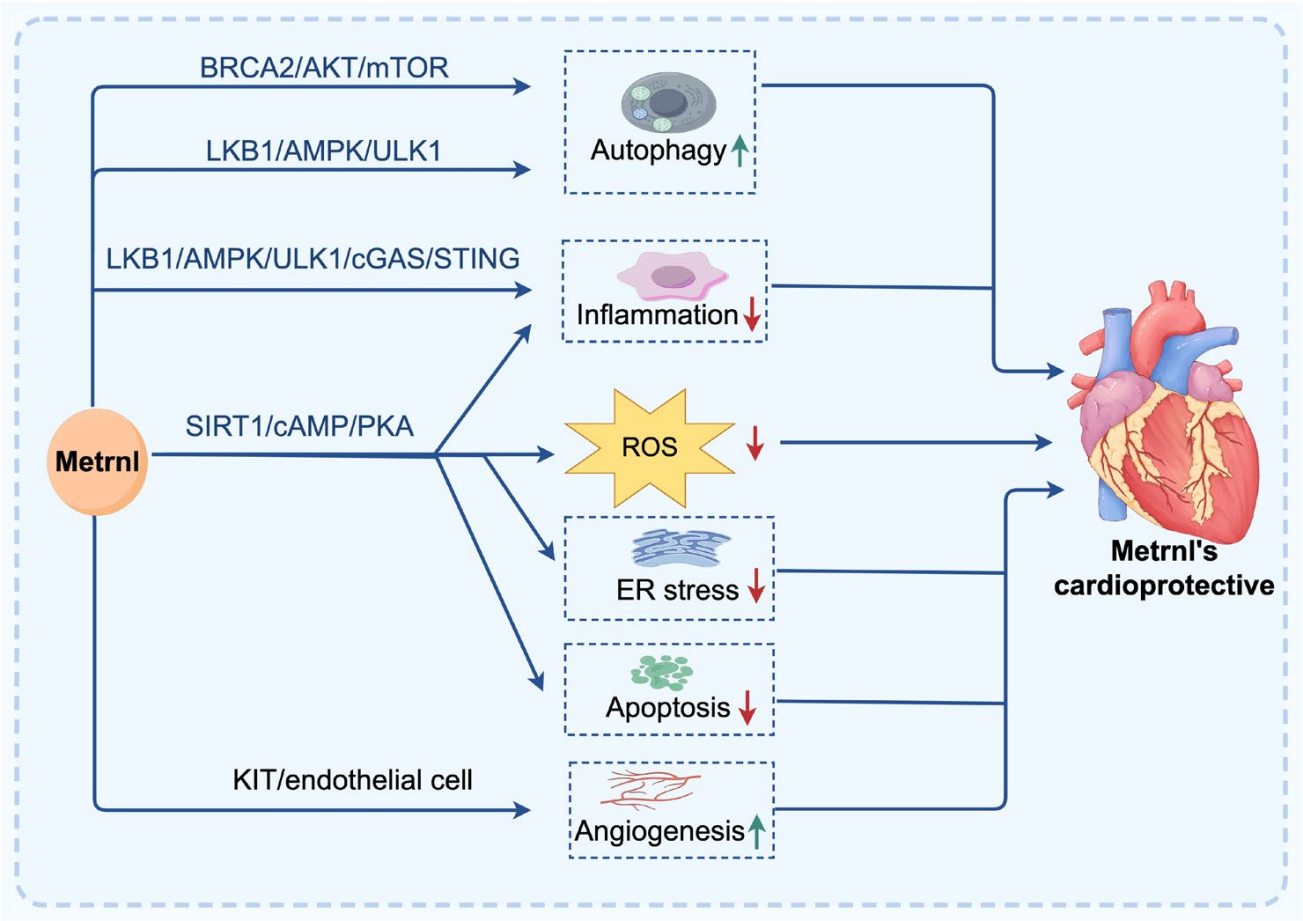
Graphical summary of the main pathways involved in Metrnl cardioprotective effects in cardiomyopathies. Figure was prepared with figdraw.com. AMPK, AMP‐activated protein kinase; BRCA2, breast cancer susceptibility gene 2; cAMP, cyclic adenosine monophosphate; cGAS, cyclic guanosine monophosphate‐adenosine monophosphate (cGAMP) synthase; LKB1, liver kinase B1; OGD, oxygen–glucose deprivation; PKA, protein kinase A; SIRT1, sirtuin 1; STING, stimulator of interferon genes; ULK1, unc‐51‐like autophagy‐activating kinase 1.

In the doxorubicin‐induced cardiotoxicity mouse model, Metrnl is downregulated, but its upregulation can be achieved, leading to improvements in oxidative stress, apoptosis and cardiac dysfunction, while its depletion exacerbates these conditions [34]. The mechanism of this process involves Metrnl activating silent information regulator sirtuin 1 (SIRT1) through the cyclic adenosine monophosphate (cAMP)/protein kinase A (PKA) pathway [34]. SIRT1, a NAD + ‐dependent deacetylase, is known for its role in promoting cellular survival and stress resistance [91]. SIRT1's interaction with the cAMP/PKA pathway could mitigate cardiotoxicity by reducing oxidative stress and inflammation in cardiac tissues. This pathway's modulation by SIRT1 may enhance the survival and function of cardiomyocytes [34, 92]. These results suggest Metrnl as a potential therapeutic strategy for DOX‐induced cardiotoxicity (Figure 2).

### 3.2 Metrnl and Diabetic Cardiomyopathy

Metrnl has been shown to improve glucose tolerance in high‐fat‐diet‐induced obesity and type 2 diabetes in mice [48]. Metrnl alleviates lipid accumulation by modulating mitochondrial homeostasis in diabetic nephropathy [93]. Although it remains unclear whether Metrnl exerts protective effects in diabetic cardiomyopathy, a condition with high global incidence. Studies in diabetic cardiomyopathy mouse models show that cardiac‐specific overexpression of Metrnl improves cardiac injury and dysfunction, while its deletion exacerbates these conditions [88]. Metrnl activates the liver kinase B1 (LKB1)/AMPK/unc‐51‐like autophagy‐activating kinase 1 (ULK1) signalling pathway, promoting autophagy and inhibiting the cyclic guanosine monophosphate‐adenosine monophosphate (cGAMP) synthase (cGAS)/stimulator of interferon genes (STING) pathway, thereby reducing myocardial injury [88]. Activation of the cGAS/STING pathway has been shown to promote cardiac fibrosis, hypertrophy and contractile dysfunction in animal models of diabetic cardiomyopathy [94, 95]. Inhibition or genetic deletion of cGAS or STING has been demonstrated to attenuate the development of diabetic cardiomyopathy and improve cardiac function in these models [96, 97].

### 3.3 Metrnl and Myocardial Infarction

Metrnl is a key regulator of angiogenesis. Metrnl deficiency was found to inhibit skin wound healing in mice by reducing angiogenesis [98], whereas topical administration of Metrnl accelerates wound epithelialization and angiogenesis in mice [99]. Angiogenesis is a crucial part of the body's natural response to a myocardial infarction [71, 100]. In mouse models of myocardial infarction, Metrnl‐deficient mice exhibit larger infarct scars, more pronounced left ventricle dilatation and greater contractile dysfunction compared with their wild‐type littermates [64]. To explore the mechanism involved in this process, endothelial cell proliferation and migration assays were conducted after scratch injury. The results showed that Metrnl promotes angiogenesis by selectively expanding the stem cell factor receptor KIT (KIT)‐expressing endothelial cell population [64]. Activating mutations in the KIT proto‐oncogene can lead to increased KIT receptor signalling and contribute to tumour‐driven angiogenesis [101, 102]. Inhibitors of the KIT receptor tyrosine kinase, such as sunitinib, have been shown to diminish angiogenesis by blocking KIT signalling [103, 104, 105]. Together, these results highlight Metrnl's crucial role in promoting angiogenesis in ischemic tissue repair.

### 3.4 Metrnl and Myocardial Hypertrophy

Metrnl is proposed as a potential therapeutic target for myocardial hypertrophy. In spontaneously hypertensive rats (SHRs), which exhibit hypertension and myocardial hypertrophy, cardiac‐specific overexpression of Metrnl can partially ameliorate these conditions [62]. Metrnl has also been found to suppress Angiotensin II (Ang II)‐induced autophagy in H9c2 cardiomyocytes by regulating the Breast Cancer susceptibility gene 2 (BRCA2)/Akt/mTOR signalling pathway. The knockdown of BRCA2 expression negates this effect [62].

In addition, the effects of Metrnl on pathological cardiac hypertrophy were explored. Overexpression of Metrnl was found to ameliorate the pathological cardiac hypertrophy induced by transverse aortic constriction both in the mouse model and in neonatal cardiomyocytes. The cardioprotective effects of Metrnl were linked to the activation of AMPK and SIRT1 [106]. Activation of the SIRT1 pathway can inhibit pathological myocardial remodelling in a TAC‐induced cardiomyocyte hypertrophy model [107]. In summary, these studies suggest that Metrnl has potential as a therapeutic target for treating myocardial hypertrophy through its effects on BRCA2/Akt/mTOR and AMPK/SIRT1 signalling pathways in cardiomyocytes.

### 3.5 Metrnl and Myocardial Ischemia/Reperfusion Injury

Oxygen–glucose deprivation (OGD) followed by reperfusion is a widely used in vitro model to study the specific features of ischemia–reperfusion injury in cardiomyocytes and other cell types [108, 109, 110]. The expression of Metrnl was found to be downregulated in H9C2 cells during OGD/R. Overexpression of Metrnl inhibited the secretion of inflammatory cytokines (including tumour necrosis factor‐alpha, IL‐1β and IL‐6), apoptosis, and endoplasmic reticulum (ER) stress, and promoted the activation of the AMPK‐PAK2 signalling cascade in H9C2 cells during OGD/R [63]. Activation of the AMPK‐PAK2 signalling pathway can reduce cardiomyocyte death and suppress ER stress induced by hypoxia‐reoxygenation injury [111, 112, 113]. The protective role of Metrnl was diminished by PAK2 silencing in H9C2 cells during OGD/R [63]. Although there are no in vivo studies confirming the protective effects of Metrnl in myocardial ischemia/reperfusion injury, Metrnl could serve as a potential therapeutic strategy for this condition.

## 4 Gaps and Future Perspective

Despite the promising study, further research is required to fully realise Metrnl's therapeutic potential in cardiomyopathies. Detailed exploration of Metrnl's signalling pathways and interactions with other molecular players in cardiomyopathies is essential. Rigorous clinical trials are needed to evaluate the safety, efficacy and optimal dosing of Metrnl‐based therapies in human subjects. Investigating Metrnl as a biomarker for early diagnosis, prognosis and monitoring therapeutic responses in cardiomyopathy patients could enhance clinical management and personalised treatment approaches. Exploring the synergistic effects of Metrnl with existing pharmacological agents or other emerging therapies could offer comprehensive benefits and improve patient outcomes. By addressing these research avenues, we can advance the development of innovative and precise therapeutic strategies that leverage Metrnl's cardioprotective properties, ultimately improving outcomes for patients with cardiomyopathies.

## 5 Conclusion and Future Perspective

In summary, Metrnl, a myokine, adipokine and cardiokine, plays critical roles in ameliorating cardiomyopathy pathogenesis through mechanisms such as reducing oxidative stress, inflammation and apoptosis, while enhancing autophagy, angiogenesis and metabolic regulation. Notably, Metrnl has shown promise in improving outcomes in models of doxorubicin‐induced cardiotoxicity, diabetic cardiomyopathy, myocardial infarction, myocardial hypertrophyss and myocardial ischemia/reperfusion injury. These findings suggest that Metrnl could be harnessed to develop more precise and effective treatments for cardiomyopathies.

## Author Contributions

**Miaomiao Xu:** writing‐review and visualisation original draft; **Xiaoguang Liu:** writing‐review and visualisation original draft; **Liming Lu:** writing‐review‐conceptualization; **Zhaowei Li:** writing‐review‐conceptualization.

## Conflicts of Interest

The authors declare no conflicts of interest.

## Data Availability

Data sharing not applicable to this article as no datasets were generated or analysed during the current study.
